# MicroRNAs as potential diagnostic markers of glial brain tumors

**DOI:** 10.1016/j.ncrna.2022.09.008

**Published:** 2022-09-22

**Authors:** Albert Sufianov, Sema Begliarzade, Tatiana Ilyasova, Xun Xu, Ozal Beylerli

**Affiliations:** a Department of Neurosurgery, Sechenov First Moscow State Medical University (Sechenov University), Moscow, Russia; b Republican Clinical Perinatal Center, Ufa, Republic of Bashkortostan, 450106, Russia; c Department of Internal Diseases, Bashkir State Medical University, Ufa, Republic of Bashkortostan, 450008, Russia; d Department of Neurosurgery, The First Affiliated Hospital of Harbin Medical University, Harbin, 150001, China; e Educational and Scientific Institute of Neurosurgery, Рeoples’ Friendship University of Russia (RUDN University), Moscow, Russia

**Keywords:** Brain tumors, Glioblastomas, miRNAs, Molecular markers, Diagnostic markers

## Abstract

Gliomas are the most invasive brain tumors characterized by high mortality and recurrence rates. Glioblastoma (GBM), a grade IV brain tumor, is known for its heterogeneity and resistance to therapy. Modern diagnostics of various forms of malignant brain tumors is carried out mainly by imaging methods, such as magnetic resonance imaging, electroencephalography, positron emission tomography, and tumor biopsy is also used. The disadvantages of these methods are their inaccuracy and invasiveness, which entails certain risks for the patient's health, so modern science has stepped up the search for more reliable and safe methods for diagnosing gliomas, including the search for novel biomarkers. MicroRNA (miRNAs), a class of small non-coding RNA, perform the most important functions in various biological processes. In recent years, great progress in the study of miRNAs paths associated with the GBM pathogenesis has been achieved. MiRNAs molecules were identified as diagnostic and prognostic biomarkers, and can also serve as therapeutic targets and agents. This review provides current knowledge about the role of miRNAs in the pathogenesis of glial brain tumors, as well as the potential use of miRNAs as diagnostic and therapeutic targets for gliomas.

## 1 Introduction

Gliomas are malignant tumors of the central nervous system that originate from glial cells: astrocytes, oligodendrocytes, ependymocytes, and are divided into astrocytomas, oligodendrogliomas, ependymomas, glioblastomas, and some others [1]. The annual incidence of gliomas worldwide is approximately 6 cases per 100,000 people [2]. According to the degree of malignancy and aggressiveness, the World Health Organization divides tumors of the central nervous system into 4°, while tumor cells of the 4th degree are characterized by anaplasia, high mitotic activity, microvascular proliferation and (or) necrosis and are the most aggressive and malignant [3]. The median survival of patients varies depending on the degree of malignancy of gliomas: for diffuse IDH-mutant astrocytoma (grade 2), this indicator is 10–12 years, for glioblastoma (grade 4), on average, 10–12 months [4]. Despite the fact that measures to prevent gliomas are not yet known, the importance of their early diagnosis, as well as other types of cancer, remains obvious: an early diagnosis, before the onset of symptoms, increases the chances of successful treatment of the patient, contributing to a slowdown in the rate of growth and development. Tumors and increased survival [5]. Currently, magnetic resonance imaging (MRI) is used to diagnose brain tumors, as well as electroencephalography, if the tumor is detected when searching for the cause of epilepsy [2,6]. In addition to MRI, positron emission tomography with labeled amino acids is also used to determine the “hot spots” of metabolism and the site of biopsy taking [7]. However, recently, new methods for diagnosing gliomas have been actively studied and proposed, not only with the help of imaging and biopsy, but also by analyzing biological fluids, mainly blood and cerebrospinal fluid. New methods still need detailed study, but nevertheless, some of their advantages have already been established. Usually, to determine the type of malignant lesion, the patient must undergo surgery, which is accompanied by the risk of postoperative complications, and further observation of the tumor using MRI does not distinguish tumor progression from radiation necrosis [8]. Cancer diagnostics using blood and CSF biomarkers is non-invasive (thus facilitating the collection of samples for research and reducing the risk of adverse effects to a minimum), the ability to track tumor progression in real time, and distinguish between progression and pseudoprogression [9]. In connection with the foregoing, it remains relevant to study the current state of the problem of searching for potential biomarkers of malignant brain tumors (gliomas) in body fluids, in particular, in cerebrospinal fluid and blood. This review contains the results of research by foreign and domestic authors on the development of new methods for diagnosing gliomas. A biomarker is a biological indicator of a pathogenic process or a pharmacological response to therapy, quantified or objectively determined [10]. With the help of diagnostic biomarkers, it is possible to detect or confirm the presence of a disease, to establish a subtype of pathology [11]. The content of tumors, metabolic products of malignant cells enter the blood and cerebrospinal fluid, so their quantitative and qualitative determination in these fluids is a diagnostic criterion. In theory, proteins and individual amino acids, microRNAs, extracellular nucleic acids, exosomes, and circulating tumor cells can act as diagnostic biomarkers of glioma [12].

## 2 Involvement of micrornas in the oncogenesis of brain gliomas

**Fig. 1:**
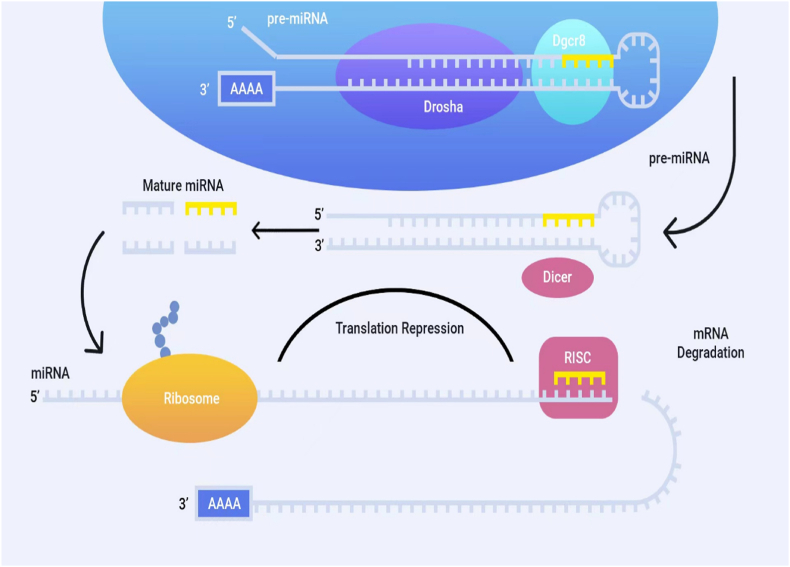
Two mechanisms used by miRNAs to regulate translation. Some miRNAs are able to bind completely complementary to target mRNAs, which causes their degradation. RISC - RNA induced silencing complex.

MicroRNAs are short non-coding RNAs, about 20–22 nucleotides long, which are involved in the control of the expression of protein-coding genes (Fig. 1) [12].

They can act as tumor growth suppressors; they can also act as oncogenes. MicroRNA regulates a large number of processes in the human body. Cell growth, its proliferative activity, tumor invasion, its metastasis, apoptosis, angiogenesis, and immune response - all these processes are regulated by microRNA [ [13,14]]. Currently, there are a number of studies of the role of individual microRNAs in brain gliomas, which indicate their diagnostic significance not only for detecting gliomas, but also in determining the degree of their malignancy. Research methods used to determine the level of microRNA expression in most studies include: real-time PCR, digital drop PCR, microarrays, sequencing. Unfortunately, the works published to date do not contain a clear systematization of the results with a description of miRNA profiles corresponding to different histotypes of gliomas and their degree of malignancy. Therefore, in our article, we will try to carry out a systematic analysis of published data on the expression of the most studied miRNAs. From the literature data, it is known that in the tissues of low-grade gliomas, compared with the surrounding brain tissue, there are multidirectional trends: along with a decrease in the expression levels of oncosuppressor miRNAs-7, -137, −153, −181, −128 there is an increase in these parameters in miRNA-9 (Table 1) [[15], [16], [17], [18]]. For oncogenic, microRNA-21 and -221/222, there is a correlation between the level of their expression in tumor tissues and the degree of malignancy of gliomas [19,20]. However, statistically significant differences that make it possible to differentiate Grade I from Grade II gliomas in terms of miRNA expression levels have not yet been obtained [20]. In high-grade gliomas, compared with paratumorous tissue, similar trends were noted: a decrease in the expression levels of oncosuppressor microRNAs-7, -31, −137, −153, −181, −128, −124 [ [19,21,22]] and, conversely, an increase in these indicators of oncogenic miRNAs-21, -23a, −221/222 [ [19,23]]. At the same time, the expression level was significantly higher in glioblastoma tissues compared to Grade III gliomas. The same pattern, but without significant differences, was noted for miRNA-9 in Grade III and Grade IV gliomas [18]. According to the levels of expression of some miRNAs, gliomas of different grades of malignancy not only differ from paratumorous tissue, but also from each other. For example, in some cases, oligodendroglioma can be distinguished from glioblastoma. In the latter, the expression levels of miRNA-21, -132, −134, −155, −218, and -409-5p are three times and miRNA-128 four times higher than in oligodendrogliomas [24].

**Table 1:** Functions of miRNAs in gliomas.

miRNAs	Function	Ref.
microRNA-221/222 (oncogene)	Proliferation (+), invasion (+), apoptosis (−), temozolomide resistance (+), radioresistance (+)	19
microRNA-23a (oncogene)	Proliferation (+), invasion (+), apoptosis (−), migration (+)	21
microRNA-21 (oncogene)	Proliferation (+), migration (+), invasion (+), apoptosis (−), temozolomide resistance (+), radioresistance (+)	19
miRNA-181 (oncosuppressor)	Invasion (−), apoptosis (+), radioresistance (+)	16, 19
miRNA-9 (oncosuppressor)	Migration (−/+), proliferation (±), radioresistance (+), growth of tumor cells (−)	18, 26, 27
miRNA-137 (oncosuppressor)	Proliferation (−), migration (−), invasion (−)	15
miRNA-124 (oncosuppressor)	Angiogenesis (−), invasion (−), metastasis (−), cell cycle (−)	15
miRNA-128 (oncosuppressor)	Proliferation (−), angiogenesis (−), apoptosis (+), differentiation (+)	17
miRNA-31 (oncosuppressor)	Proliferation (−), invasion (−)	19
miRNA-153 (oncosuppressor)	Proliferation (−), apoptosis (+)	22
miRNA-7 (oncosuppressor)	Migration (−), invasion (−), radioresistance (−)	19, 21

## 3 The role of micrornas in the development of brain gliomas

**Fig. 2:**
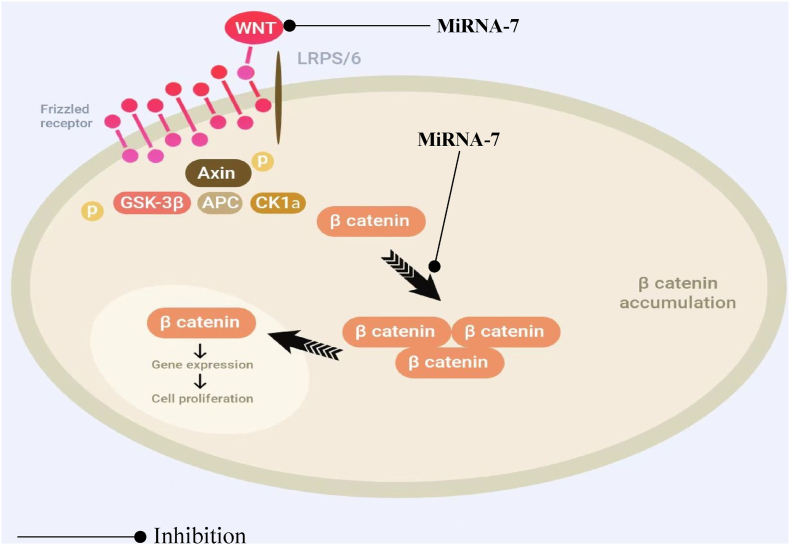
MiRNA-7 inhibits Wnt-Signaling in glioblastomas.

Oncosuppressive miRNA-7 is involved in the regulation of Wnt-dependent and Raf/Ras/ERK/MEK-dependent signaling pathways, controlling the processes responsible for tumor growth and malignancy: invasion, proliferation, migration, and apoptosis (Fig. 2).

Visani M. et al. recorded a decrease in the expression of this miRNA in gliomas compared to paratumorous tissue [21]. The level of oncosuppressive microRNA-9 was significantly increased in groups of grades I-III compared with conditionally normal adjacent brain tissues, but no statistically significant differences were found for glioblastomas [18]. Increased expression of miRNA-9 leads to a significant increase in cell migration in the tumor tissue and prevalence over their proliferation, this is associated with a decrease in the expression of NF1 and CREB. On the contrary, a decrease in miRNA-9 expression leads to a predominance of cell proliferation over their migratory ability [25]. The work of Gomez G. et al. showed that the target for oncosuppressive miRNA-9 is the FOXP1 gene. An increase in its expression level in vitro in U251 and U373 cell cultures stimulated their growth [26]. With an increase in miRNA-9 expression, resistance to chemotherapy (temozolomide) increases, this is associated with activation of the expression of the components of the SHH complex of multiple drug resistance [27].

**Fig. 3:**
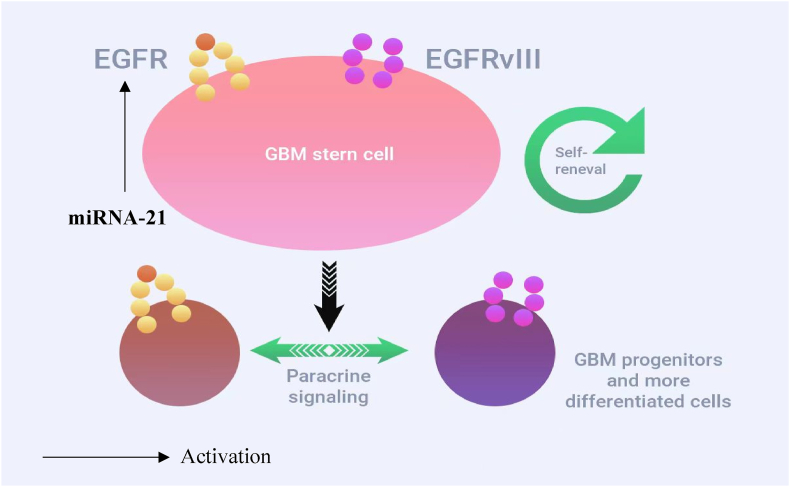
MiRNA-21 activates EGFR and EGFRvIII in glioblastomas.

MicroRNA-21 is one of the most highly expressed microRNAs in human cells; however, its expression level is further increased in glioma tissues [19]. The results of many studies of miRNA-21 indicate an increase in the level of expression in glioma tissues by about 5–15 times compared with the norm [ [19,21]]. The oncogenic effect of microRNA-21 in human glioma cells is mediated through suppression of the expression of tumor suppressor genes such as HNRPK, TAp63, JMY, RECK, TOPORS, TP53BP2, DAXX, TGFBR2/3, PDCD4, and TIMP3 [28]. An increase in miRNA expression leads to an increase in proliferation, invasion, and a decrease in apoptosis of tumor cells. In addition, the low level of its expression, according to the Cancer Genome Atlas (TCGA), is weakly associated with increased survival. Inhibition of miRNA-21 leads, along with a decrease in EGFR expression, to arrest of the cell cycle in the G1/S phase and, ultimately, to inhibition of tumor growth (Fig. 3) [29].

In high-grade gliomas, there is an increased level of expression of oncogenic microRNA-23, which, due to inhibition of the expression of the transcription factor HOXD10, which regulates the expression of MMP-14, leads to the activation of glial cell invasion [[23], [30]]. Tan X. et al. noted that an increase in the growth of gliomas is associated with increased levels of miRNA-23a expression, which depends on the transcription factor CREB and suppresses the expression of the PTE gene [31]. A decrease in the expression levels of miRNA-23a and miRNA-21 in vitro in the U138 glioma cell line reduced the ability of the tumor to form colonies [23]. MicroRNA-137 is oncosuppressive, its expression is significantly reduced not only in oligodendroglial tumors II-III, but also in grade III-IV gliomas [ [15,32]]. Visani M. et al. also obtained similar results, but a significant difference was found in gliomas only between grades I and IV [33]. As the degree of malignancy of gliomas increases (especially in glioblastomas), miRNA-137 expression levels decrease, leading to activation of proliferation and invasion of tumor cells [34]. According to Xu J. and colleagues, it was found that the expression level of microRNA-153-3p in all groups of glial tumors, compared with the control group, was significantly reduced [22]. Its target is the anti-apoptotic proteins Bcl-2 and Mcl-1.

Gliomas of low and high malignancy are characterized by activation of anti-apoptotic mechanisms and signaling pathways that enhance the survival of their cells. Along with this, microRNA-153-3p suppresses the expression of the IRS-2 activator of the PI3K/AKT-dependent signaling pathway, which is also responsible for the survival of these tumor cells. This indicates the antitumor role of microRNA-153-3p in the development of these neoplasms [35]. MicroRNA-181a and microRNA-181b are oncosuppressive, and deregulation of their expression contributes to the manifestation of glioma malignancy [27]. The target of miRNA-181a and miRNA-181b is the BCL2 gene [ [19,36]]. Shi L. et al. noted a decrease in the level of expression of these microRNAs in glioblastoma multiforme, as well as a negative correlation with the grade of glioma malignancy, so that the lowest expression values are achieved in grade II-IV gliomas [16]. Our studies have also shown that the expression level for miRNA-181b in glioma tissues is lower than in paratumorous brain tissue. Moreover, its decrease is proportional to the increase in the degree of malignancy of neoplasms [20]. The p27 and p57 proteins, as well as the PTEN, TIMP3, PUMA, and Cx43 genes, which regulate the cell cycle and cell survival processes, are targets of the oncogenic microRNA-221/222 cluster [37]. In a number of studies, an increased expression of microRNA-221, -222 in gliomas was noted [ [38,39]], which promotes cell migration by downregulating PTPμ expression, as well as increasing sensitivity to chemotherapy drugs by reducing the level of MGMT expression [40]. We have found that in the tissues of high-grade gliomas there is a statistically significant increase in the expression level of miRNA-221 compared with conditionally normal adjacent brain tissues [20]. MiRNA-128 is a neuron-specific miRNA involved in neural differentiation in malignant gliomas such as glioblastoma. It acts as an oncosuppressor [ [17,41]]. The target of this miRNA is the transcription factor E2F3a; a decrease in its expression level inhibits tumor cell proliferation and the cell cycle [42]. Zhang Y. et al. showed in their works that the level of its expression in gliomas is reduced compared to the level in paratumorous tissue [ [17,42]]. MicroRNA-124 is oncosuppressive; it is involved in neuronal differentiation. It was found that the level of its expression is reduced not only in glioblastomas and oligodendrogliomas, but also in medulloblastomas [[43], [44], [45]]. The expression level of miRNA-124 is statistically significantly reduced in gliomas (Grade III) and glioblastomas compared with paratumorous tissue [20]. It regulates the cell cycle in the G0/G1 phase and also inhibits CDK6 kinase, which stimulates angiogenesis [ [15,28]], leading to the emergence of newly formed vessels. The latter play a leading role in the further growth of neoplasms and their metastasis [46]. Transfection of miRNA-124 into glioma cell lines leads to a decrease in cell migration [47].

When studying the expression levels of miRNA-31 in the tissues of high-grade gliomas, a statistically significant decrease was found in the tumor tissue compared to the conditionally normal adjacent brain tissues [20]. In glioma cell culture, this miRNA not only inhibits cell migration and indirectly affects the activation of the transcription factor NF-kB, angiogenesis, but also the level of E-cadherin associated with the epithelial-mesenchymal transition [ [48,49]]. MicroRNA-31 acts as an oncosuppressor, and its level can correlate with the predisposition of the tumor to invasion and metastasis [20].

## 4 Conclusions

To date, extensive experience has been accumulated in determining the level of microRNA expression in human brain glioma tissues using various methods [[50], [51], [52], [53], [54], [55], [56]]. But there are certain obstacles that limit the application of these methods when using miRNAs in clinical diagnostics. Firstly, this is due to the fact that many studies have been performed on small groups of patients with different histological structures of brain tumors. Secondly, brain gliomas are heterogeneous neoplasms, which complicates the interpretation of the obtained data and the selection of miRNAs that could act as diagnostic markers. In this regard, an important task is to standardize methods for determining miRNA expression levels and selecting optimal reference genes. To obtain reliable data, large-scale prospective studies are needed to determine the role of microRNAs in the oncogenesis of gliomas and confirm their effectiveness as biomarkers in the diagnosis of neuroepithelial brain tumors. The ongoing in-depth study of the molecular genetic characteristics of gliomas based on the study of the role of miRNAs in oncogenesis is, in our opinion, a promising direction in determining the molecular mechanisms of tumor growth for prescribing timely and adequate adjuvant therapy in complex treatment, as well as predicting the course of oncological disease.

## Funding

This study was supported by the Bashkir State Medical University Strategic Academic Leadership program (PRIORITY-2030).

## Author contributions

Albert Sufianov and Sema Begliarzade conceptualized and designed the study. All authors participated in the acquisition, analysis and interpretation of the data. Tatiana Ilyasova and Xun Xu drafted the manuscript. Ozal Beylerli contributed to critical revisions of the manuscript. All authors agreed on the journal to which the article would be submitted, gave final approval for the version to be published, and agreed to be accountable for all aspects of the work.

## Declaration of competing interest

The authors declare they have no conflict of interest.
